# Performance evaluation of three i‐SENS glucometers using arterial blood samples compared with the YSI 2300 Glucose Analyzer

**DOI:** 10.1002/jcla.23356

**Published:** 2020-05-19

**Authors:** Ha Nui Kim, Kyung Chul Moon, Soo‐Young Yoon

**Affiliations:** ^1^ Department of Laboratory Medicine Korea University Guro Hospital Seoul Korea

**Keywords:** arterial blood i‐SENS, glucometer, POCT, point‐of‐care

## Abstract

**Background:**

Capillary blood is the most commonly used sample for point‐of‐care (POC) glucometers. However, in critically ill patients, the glucose levels measured from capillary blood may not be reliable. Thus, we aimed to evaluate and compare the accuracy of glucose levels measured with POC glucometers and the YSI 2300 glucose analyzer using leftover arterial blood samples.

**Methods:**

In total, 100 leftover heparinized arterial blood samples were used to evaluate the performance of three i‐SENS glucometers (BAROzen H Expert plus, CareSens PRO, and CareSens H Beat) and the ACCU‐CHEK^®^ Inform II glucometer. The reference value was obtained using the YSI 2300 glucose analyzer. The results were analyzed based on International Organization for Standardization 15197:2013 guidelines.

**Results:**

More than 95% of results obtained using POC glucometers were within ±15 mg/dL of the reference value for glucose concentrations <100 mg/dL and within ±15% of the reference value for glucose concentrations ≥100 mg/dL. In the consensus error grid analysis, more than 99% of results were found to be within zones A and B. An excellent correlation was found between the values obtained using POC glucometers and the YSI 2300 glucose analyzer (*R* ^2^ > .99).

**Conclusion:**

The i‐SENS glucometers showed stable and accurate results when leftover arterial blood samples were used. Therefore, POC glucometers could be useful in critical care settings, such as intensive care units, where arterial samples are routinely used.

## 1 INTRODUCTION

Currently, self‐monitoring of blood glucose (SMBG) is widely used for patients with diabetes as well as for those in a critical condition. Both hyperglycemia and hypoglycemia adversely affect critically ill patients. Tight glucose control for preventing blood glucose fluctuation has significant survival benefits for critically ill patients.^1^ For blood glucose measurement, capillary blood samples (via glucometers) and venous or arterial whole blood samples (via central laboratory devices) are routinely used. The dependency of the physiological activity of glucose on its plasma concentration varies with hematocrit levels. Thus, central laboratory measurements obtained using plasma from venous blood are recommended.^2^ In critical care settings, plasma measurements are not suitable owing to time constraints. Therefore, point‐of‐care (POC) testing devices, including glucometers and blood gas analyzers, are commonly used in intensive care units (ICUs).^3^

Although POC glucometers are widely used in various hospital settings to examine capillary blood specimens, a recent multi‐center study revealed the growing concerns in ICU clinicians regarding the inaccuracy of glucometers.^4^ Results obtained from glucometers may be inaccurate owing to hematocrit interference and oxygen effects in glucose oxidase (GOD)‐based devices. Recently developed glucometers use a glucose dehydrogenase (GDH)‐based system, which is not affected by oxygen levels and also minimizes hematocrit interference by applying an algorithm that converts an internal signal into a measured value. In the absence of such interference, POC glucometers provide better speed and simplicity than do blood gas analyzers. Usually, arterial blood samples are obtained regularly from patients in the ICU in order to monitor their oxygen levels. We therefore aimed to evaluate the accuracy of arterial blood measurements obtained from POC glucometers using a reference measurement system, the YSI 2300 STAT Plus glucose analyzer (YSI 2300, Yellow Springs Instrument Inc.). Here, we present the performances of three glucometers manufactured by i‐SENS Inc., which are designed to minimize the effects of hematocrit and oxygen levels, compared with that of the YSI 2300 Stat Plus Analyzer in evaluating blood glucose levels using arterial blood samples. We also performed a brief study using the ACCU‐CHEK^®^ Inform II Blood Glucose Meter (Roche Diagnostics), which is widely used worldwide.

## 2 MATERIALS AND METHODS

### 2.1 Samples

We retrospectively evaluated 100 heparinized arterial blood samples obtained between January 2019 and March 2019. The samples were originally collected for arterial blood gas analysis (ABGA) from patients who visited the Korea University Guro Hospital. Specimens remaining after ABGA were used to evaluate the performance of three i‐SENS glucometers, as well as that of the ACCU‐CHEK^®^ Inform II glucometer and the YSI 2300 glucose analyzer. We excluded samples showing hemolysis or contamination and those with volume <350 µL or hematocrit levels out of 15‐65%. All samples were handled anonymously and were assigned a new identification number. This study was approved by the Institutional Review Board of Korea University Guro Hospital (IRB No: 2019GR0016).

### 2.2 i‐SENS glucometers

Three commercially available i‐SENS glucometers (BAROzen H Expert plus, CareSens PRO, and CareSens H Beat) from Korea were evaluated in this study. BAROzen H Expert plus is used in medical institutions, while the other two glucometers are used for SMBG in general diabetes healthcare. Apart from this, there are no significant differences in the methodology of these glucometers. All i‐SENS glucometers use a GDH system, while the ACCU‐CHEK^®^ Inform II glucometer uses mutant quinone GDH in order to prevent maltose interference.

### 2.3 ISO 15197:2013 guideline

Evaluations were performed according to the International Organization for Standardization (ISO) 15197:2013, the guideline for requirements of glucometer performance. For a glucometer to be considered accurate, ISO 15197:2013 recommends that^1^: Compared with the results of a central laboratory method, at least 95% of glucometer results have to be within ±15 mg/dL at glucose concentrations <100 mg/dL and within ±15% at glucose concentrations ≥100 mg/dL; and^2^ in a consensus error grid analysis, at least 99% of glucometer results have to be within zones A and B.^5^ In our study, we set the arterial blood glucose value measured by the YSI 2300 glucose analyzer as the reference value and examined whether the three i‐SENS glucometers fulfilled the ISO 15197:2013 criteria.

### 2.4 Study protocols

The samples examined in this study were allowed to rest at room temperature before measurement; analyses were performed within 30 minutes for each sample. Three different lots were prepared for each i‐SENS glucometer, and one lot was prepared for ACCU‐CHEK^®^ Inform II. Hematocrit levels were determined using a Sorvall Legend Micro 17 centrifuge (Thermo Fisher Scientific). Whole blood glucose levels were measured using the YSI 2300 glucose analyzer and converted to plasma glucose values using a preprogrammed algorithm that incorporated the given hematocrit level.^6^ Using an arterial blood gas analyzer (ABL FLEX 700; Radiometer), the oxygen level in the samples was also measured, in order to exclude unsuitable specimens. Glucose levels were simultaneously measured using the three i‐SENS glucometers (BAROzen H Expert plus, CareSens PRO, and CareSens H Beat) and ACCU‐CHEK^®^ Inform II. Three different strips (lot A, B, and C) for i‐SENS glucometers and one strip for ACCU‐CHEK^®^ Inform II were inserted into the glucometer. Each strip loaded with 0.4 µL (0.5 uL for CareSens H Beat) of arterial blood and all measurements were performed twice. The difference between the reference value (from the YSI 2300 glucose analyzer) and the value obtained from each glucometer was calculated; Passing‐Bablok regression analysis was used to determine the correlation between measured values. All statistical analyses were performed using Microsoft Excel 2016 (Microsoft, NY, USA) and Analyse‐it (Analyse‐it Software Ltd., The Tannery).

## 3 RESULTS

The distribution of blood glucose concentrations measured using the YSI 2300 glucose analyzer, as defined by ISO 15197:2013, is presented in Table 1. There were insufficient numbers of samples in the low (<80 mg/dL; n = 8) and high (>200 mg/dL; n = 21) blood glucose concentrations that could not meet the requirement in ISO 15197:2013.

**Table 1:** Distribution of blood glucose concentrations

Glucose concentrations (mg/dL)	Our study (%)	ISO 15197:2013 recommendation (%)
≤50	2	5
>50‐80	10	15
>80‐120	42	20
>120‐200	37	30
>200‐300	6	15
>300‐400	1	10
>400	2	5

**Figure 1:**
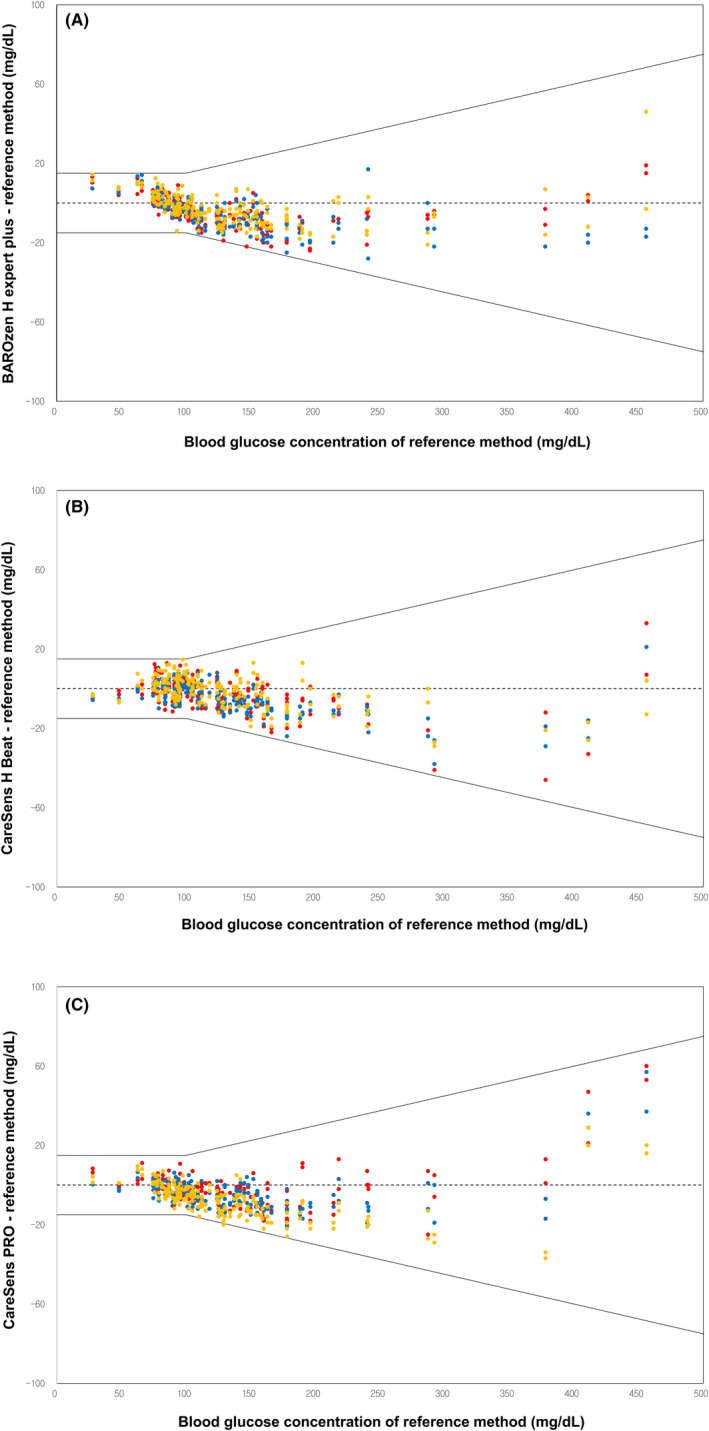
Difference‐plots of i‐SENS glucometers obtained from the results of three different lots (yellow dots: lot No. QL26LBB1A, blue dots: lot No. QL26LBB3G, red dots: lot No. QL31LBA9A)

With respect to the ISO 15197:2013 criteria, data from three different lots for i‐SENS glucometers and one lot for ACCU‐CHEK^®^ Inform II—measured twice without averaging—are summarized in Table 2. Difference plots containing all results from all lots and comparing the reference value with values obtained using the three i‐SENS glucometers are displayed in Figure 1. All results from the BAROzen H Expert plus and CareSens H Beat were within the acceptable range, while 99.8% (599/600) of results from the CareSens PRO were within the acceptable range (Figure 1C). A slight negative bias is observed in difference plots according to the fact that the whole blood glucose levels are usually lower than the plasma glucose level by 10%‐15%.

**Table 2:** Accuracy of i‐SENS glucometers and ACCU‐CHEK® Inform II

Glucometers	Glucose concentrations <100 mg/dL			Glucose concentrations ≥100 mg/dL		
	Within ±5 mg/dL	Within ±10 mg/dL	Within ±15 mg/dL	Within ±5 %	Within ±10 %	Within ±15 %
BAROzen H Expert plus	169/222	210/222	222/222	173/378	337/378	378/378
	(76.1%)	(94.6%)	(100%)	(45.8%)	(89.2%)	(100%)
CareSens H Beat	148/222	209/222	222/222	218/378	363/378	378/378
	(66.7%)	(94.1%)	(100%)	(57.7%)	(96%)	(100%)
CareSens PRO	158/222	218/222	222/222	162/378	325/378	377/378
	(71.2%)	(98.2%)	(100%)	(42.9%)	(86%)	(99.7%)
ACCU‐CHEK^®^ Inform II	39/74	57/74	71/74	62/126	102/126	121/126
	(52.7%)	(77.0%)	(95.9%)	(49.2%)	(81.0%)	(96.0%)

Different ranges of glucose concentrations (±5 mg/dL or ±5% and within ±10 mg/dL or ±10%) are also stipulated in the Food and Drug Administration (FDA) guidelines. ISO 15197:2013 suggests that at least 95% of glucose values should fall within ±15 mg/dL of the reference standard at glucose concentrations <100 mg/dL and within ±15% at glucose concentrations ≥100 mg/dL.^7^ All glucometers used in our study met the ISO 15197:2013 acceptance criteria, although the proportion of values falling within the recommended range gradually decreased as the range became narrower. However, compared with the ACCU‐CHEK^®^ Inform II, all i‐SENS glucometers generally showed better performance, especially within the range of ±10 mg/dL or 10%.

The correlation between the values obtained using the POC glucometers and the YSI 2300 glucose analyzer, including the slope, *y*‐intercept, 95% confidence intervals (CI), and correlation coefficient (*R* ^2^) calculated using Passing‐Bablok regression, are summarized in Table 3. All glucometers showed excellent correlation (*R* ^2^ > .95) with the reference value. Additional comparison study between the three i‐SENS glucometers and ACCU‐CHEK^®^ Inform II also showed an excellent correlation (*R* ^2^ = .984 with BAROzen H Expert plus, *R* ^2^ = .991 with CareSens H Beat, and *R* ^2^ = .982 with CareSens PRO).

**Table 3:** Summary of regression plot of glucometers compared with reference value using YSI 2300 by Passing‐Bablok regression

Glucometer	Slope (Bootstrap 95% CI)	Intercept (Bootstrap 95% CI)	*R* ^2^
BAROzen H Expert plus	0.882 (0.866‐0.900)	9.353 (7.625‐11.12)	.994
CareSens H Beat	0.922 (0.911‐0.934)	6.376 (4.942‐8.007)	.994
CareSens PRO	0.909 (0.895‐0.923)	5.409 (3.891‐6.888)	.991
ACCU‐CHEK^®^ Inform II	0.985 (0.975‐0.996)	7.301 (5.743‐8.859)	.991

**Figure 2:**
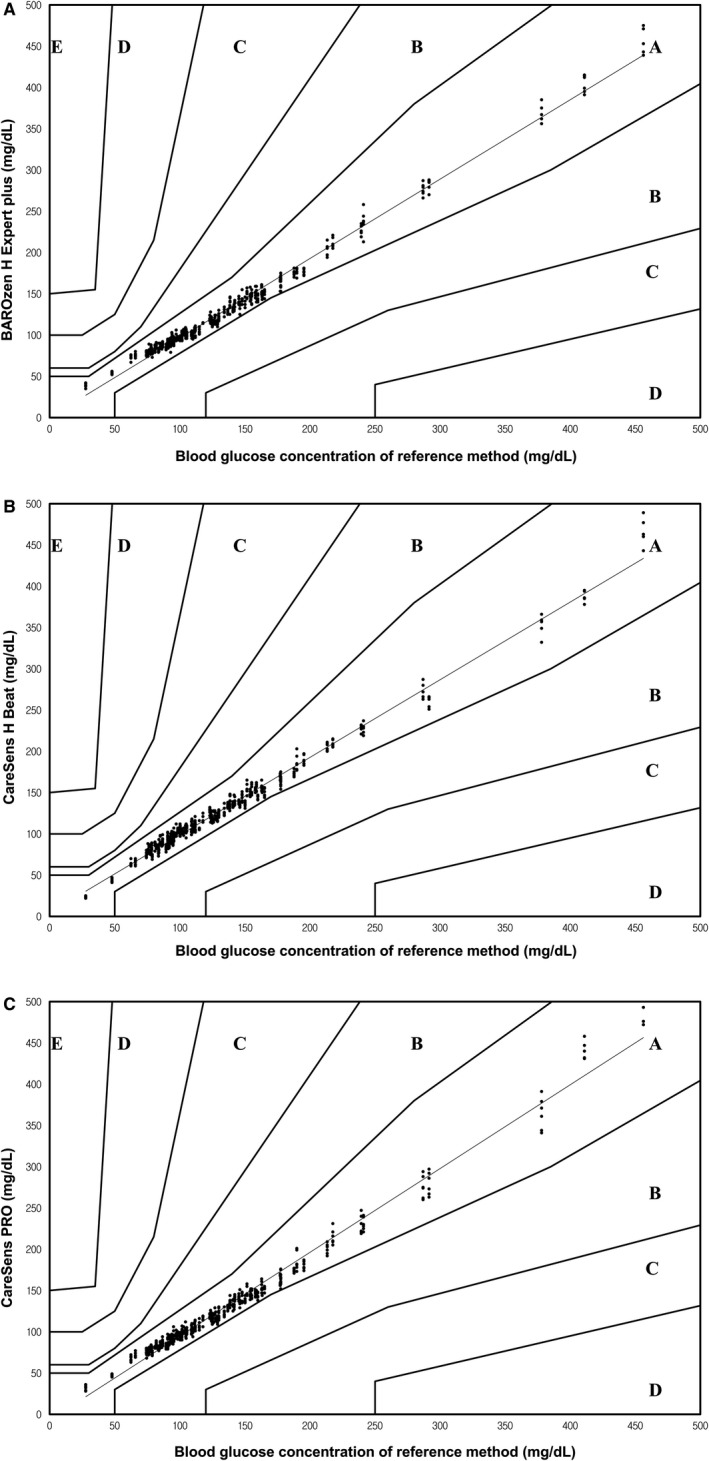
Consensus error grid analysis of three i‐SENS glucometers showing all results from three different lots. Zone A: no effect on clinical action; zone B: altered clinical action (little or no effect on clinical outcome); zone C: altered clinical action (likely to affect clinical outcome); zone D: altered clinical action (could have significant medical risk); zone E: altered clinical action (could have dangerous consequences)

The other accuracy criterion put forth by ISO 15197:2013 requires that 99% of all results should be in zones A and B of the consensus error grid. The i‐SENS glucometers showed excellent results, as the measured glucose values from the three different lots were all in zone A (Figure 2).

## 4 DISCUSSION

Our study was designed to confirm whether arterial blood was suitable for glucose measurements using commonly used blood glucometers. The laboratory measurements of blood glucose using serum or plasma are widely considered the “gold standard” but often fail to meet expectations, mainly due to time‐related delays. In contrast, POC glucometers that require capillary blood—which can be obtained by pricking the patient's finger—can be used to quickly measure the patient's glucose levels. However, in some patients with severe anemia, metabolic acidosis, and hypoxia, the results obtained from capillary blood specimens may be inaccurate owing to various sources of interference.^8^, ^9^, ^10^ Recently developed glucometers apply a unique algorithm developed by manufacturers to minimize hematocrit interference and offer stable results within a hematocrit range of 10‐70%.^11^ Nevertheless, it is recommended that arterial samples—rather than capillary blood—be used to test glucose levels in critically ill patients, and especially in patients with shock who are on vasopressor support.^12^ A systematic review showed that in critically ill patients, the results obtained with arterial blood were significantly more accurate than those obtained with capillary blood when arterial blood gas analyzers and/or glucometers were used.^13^

Arterial blood gas analysis is an essential and routine test performed to monitor oxygenation status and acid‐base balance in ICU patients.^14^ It may be beneficial to measure blood glucose levels using arterial blood samples that remain after routine ABGA. To ensure that POC glucometers provide accurate and stable results with arterial blood samples, a verification procedure is necessary. We used several types of glucometers to confirm the stability of leftover arterial blood samples in this study.

As mentioned previously, hematocrit values are known to strongly affect measured plasma glucose levels.^8^ Glucometers measure glucose in whole blood and correct the measured value using a specified formula under the assumption that the hematocrit level is normal.^8^, ^15^ Hence, in patients with anemia, hypoglycemia can go undetected due to false high glucose values and can lead to the administration of incorrect insulin dosages.^16^ Therefore, ISO 15197:2013 recommends the use of test procedures and acceptance criteria to evaluate the influence of hematocrit, which can interfere with glucose measurement. i‐SENS glucometers extend the hematocrit range, with no effect on glucose values, by applying specific algorithms that convert internal signals into measured values, similar to other recently developed glucometers that show no significant interference of hematocrit levels.^17^

The most commonly used enzymes for measuring blood glucose levels are GDH, which is used in i‐SENS glucometers, and GOD. GOD‐based glucometers are prone to oxygen interference because oxygen is a physiological electron acceptor and is naturally affected by both low and high oxygen levels. In contrast, GDH is not affected by oxygen levels because oxygen is not involved in its electrochemical reaction.^18^ In our study, we also measured oxygen levels in samples to identify any unsuitable specimens, but oxygen levels were found to have no significant effect on the results because all i‐SENS glucometers used in the study were GDH‐based.

There are some limitations to our study. First, the number of samples with low and high blood glucose concentrations was limited owing to challenges with sample collection. We could not generate the percentage of samples recommended by ISO 15197:2013 through modification because it is difficult to artificially modify the glucose concentrations in leftover arterial blood samples. The ISO 15197:2013 distribution criteria for glucose concentrations should be strictly applied when evaluating newly developed devices, but i‐SENS glucometers are already certified for blood glucose measurement and our aim was to prove that leftover arterial blood samples are also suitable for these analyses.

Second, although the results obtained from the i‐SENS glucometers and the YSI 2300 glucose analyzer using leftover arterial blood samples showed excellent agreement, we cannot verify whether the glucose levels in arterial blood samples actually correlate with the patient's plasma blood glucose levels. Since this study used residual arterial blood samples, we cannot simultaneously measure the venous glucose levels of patients at the same time. Moreover, whether the difference between arterial and venous glucose values is significant remains unclear.^19^ The current guidelines on glucose measurement in critically ill patients indicate that both arterial and venous specimens are acceptable and considered equivalent, but capillary blood specimens are not.^20^ Although no direct comparison was made due to the lack of additional venous glucose data, the merits of using arterial blood samples seem to be definite.

Previous studies have reported the inaccuracy of POC glucometers in measuring blood glucose levels using venous blood, and that only arterial blood can be used with POC glucometers for accurate blood glucose measurements.^19^ Larger differences are mainly observed in patients with hyperglycemia, low hematocrit levels, and acidosis, which are frequently observed in ICU settings. Our study showed that arterial blood samples can provide stable and accurate results with using different types of glucometers.

In conclusion, our study showed satisfactory performance of the i‐SENS glucometers compared with the reference values and comparable performance to the ACCU‐CHEK^®^ Inform II. Although there was a limitation due to the insufficient numbers of samples in low and high blood glucose concentrations, the i‐SENS glucometers presented acceptable differences and consensus error grid analysis results when compared to the reference glucose concentrations in all the measured glucose values.
